# Integrins and extracellular matrix proteins modulate adipocyte thermogenic capacity

**DOI:** 10.1038/s41598-021-84828-z

**Published:** 2021-03-08

**Authors:** Maria A. Gonzalez Porras, Katerina Stojkova, Marcella K. Vaicik, Amanda Pelowe, Anna Goddi, Alanis Carmona, Byron Long, Amina A. Qutub, Anjelica Gonzalez, Ronald N. Cohen, Eric M. Brey

**Affiliations:** 1 Department of Biomedical Engineering and Chemical Engineering, AET 1.102, The University of Texas at San Antonio, 1 UTSA Circle, San Antonio, TX 78249 USA; 2 Department of Biomedical Engineering, Illinois Institute of Technology, Chicago, IL USA; 3 Department of Biomedical Engineering, Yale University, New Haven, CT USA; 4 Section of Endocrinology, Diabetes, and Metabolism, Department of Medicine, University of Chicago, Chicago, IL USA

**Keywords:** Extracellular matrix, Integrins, Stem-cell differentiation

## Abstract

Obesity and the metabolic disease epidemic has led to an increase in morbidity and mortality. A rise in adipose thermogenic capacity via activation of brown or beige fat is a potential treatment for metabolic diseases. However, an understanding of how local factors control adipocyte fate is limited. Mice with a null mutation in the laminin α4 (LAMA4) gene (KO) exhibit resistance to obesity and enhanced expression of thermogenic fat markers in white adipose tissue (WAT). In this study, changes in WAT extracellular matrix composition in the absence of LAMA4 were evaluated using liquid chromatography/tandem mass spectrometry. KO-mice showed lower levels of collagen 1A1 and 3A1, and integrins α7 (ITA7) and β1 (ITB1). ITA7-ITB1 and collagen 1A1-3A1 protein levels were lower in brown adipose tissue compared to WAT in wild-type mice. Immunohistochemical staining confirmed lower levels and different spatial distribution of ITA7 in KO-WAT. In culture studies, ITA7 and LAMA4 levels decreased following a 12-day differentiation of adipose-derived stem cells into beige fat, and knock-down of ITA7 during differentiation increased beiging. These results demonstrate that extracellular matrix interactions regulate adipocyte thermogenic capacity and that ITA7 plays a role in beige adipose formation. A better understanding of the mechanisms underlying these interactions can be used to improve systemic energy metabolism and glucose homeostasis.

## Introduction

Obesity, a chronic imbalance in energy homeostasis between energy intake and energy expenditure, is a public health crisis and continues to be among the most important medical challenges in the U.S.A. Obesity creates a greater than $190 billion burden annually on American healthcare^1^. with the percent of US medical expenditures devoted to treating obesity increasing 29% from 2001 to 2015^2^. According to the most recent CDC statistics, over 70% of the United States population is overweight or obese, and more than 120 million people have diabetes or pre-diabetes^3,4^. The development of therapeutic approaches that lessen or eliminate obesity-associated morbidity and mortality would have a transformative impact on the American population and healthcare spending. There are two types of adipose tissue in mammals: white adipose tissue (WAT) which is specialized for energy storage, and brown adipose tissue (BAT), specialized for energy expenditure and heat generation^5–7^. The significant capacity of BAT for energy expenditure may be a mechanism for the treatment of metabolic disease, but the small volume of BAT in adults suggests that therapeutic potential is limited^8^. However, “brown-like” adipocytes have been detected in human WAT. These “brown-in-white” or beige cells can be induced in WAT and could be a more viable approach^9–11^. Engineering BAT or beige adipose tissue depots in the lab for subsequent transplantation has also been explored as a treatment for metabolic disease in pre-clinical models^12–14^. While investment has been made in designing therapeutics that target beige or brown adipose tissue, it remains difficult to maintain adipose tissue transformation in vivo. This results, in part, from our incomplete knowledge concerning the mechanisms underlying beige and brown adipose tissue formation.

Recently, cell-extracellular matrix (ECM) interactions have been shown to influence beige and brown adipose formation and function^12,15–17^. In addition, we have found that the absence of laminin α4 protein (LAMA4) results in persistent beige adipose tissue formation, resistance to obesity, and enhanced metabolic function^18,19^. Cells cultured on complex ECM exhibited increased expression of uncoupling protein 1 (UCP1), a primary marker of beige/brown adipocytes, when LAMA4 was absent^20,21^. While it is clear that the absence of LAMA4 results in increased expression of UCP1 in subcutaneous WAT (SubQ WAT) and BAT^18^, the mechanism driving this behavior is unknown.

Disrupting the expression of a single protein can result in a broad range of changes to ECM properties^22^. The composition may be altered due to compensatory changes in other ECM proteins. The disruption can influence ECM assembly resulting in changes to the structure and mechanical properties. These broad changes indicate that the UCP1 expression observed in the absence of LAMA4 may result from other changes secondary to the absence of LAMA4. The fate of adipose tissue stem cells (ASC) is influenced by both the chemical composition and mechanical properties of the ECM, and cell-ECM interactions primarily occurs through integrins^23^. Integrins are a family of transmembrane receptors consisting of 18 α-subunits and 8 β-subunits^24^ that form at least 24 heterodimers. Integrins can activate signaling pathways that influence gene expression and cell function and may be a better target for therapeutic intervention than ECM molecules. Therefore, a fundamental understanding of matrix-integrin interactions that regulate the thermogenic capacity of adipose tissues will provide new insights into adipose function while informing new therapeutic options for engineering environments that stimulate ASCs beige differentiation.

In this study, the ECM composition of adipose tissue of LAMA4 knockout mice (KO) was examined in order to identify candidate ECM molecules and integrins potentially involved in regulating UCP1 expression. The data suggests that ECM molecules present at higher levels in WAT inhibit UCP1 expression, and that disrupting integrin interactions with the ECM can increase UCP1 expression. This work provides substantial support for integrin-matrix interactions as important modulators of the thermogenic capacity of adipose tissue.

## Results

### Absence of laminin α4 alters ECM composition and integrin levels

**Figure 1:**
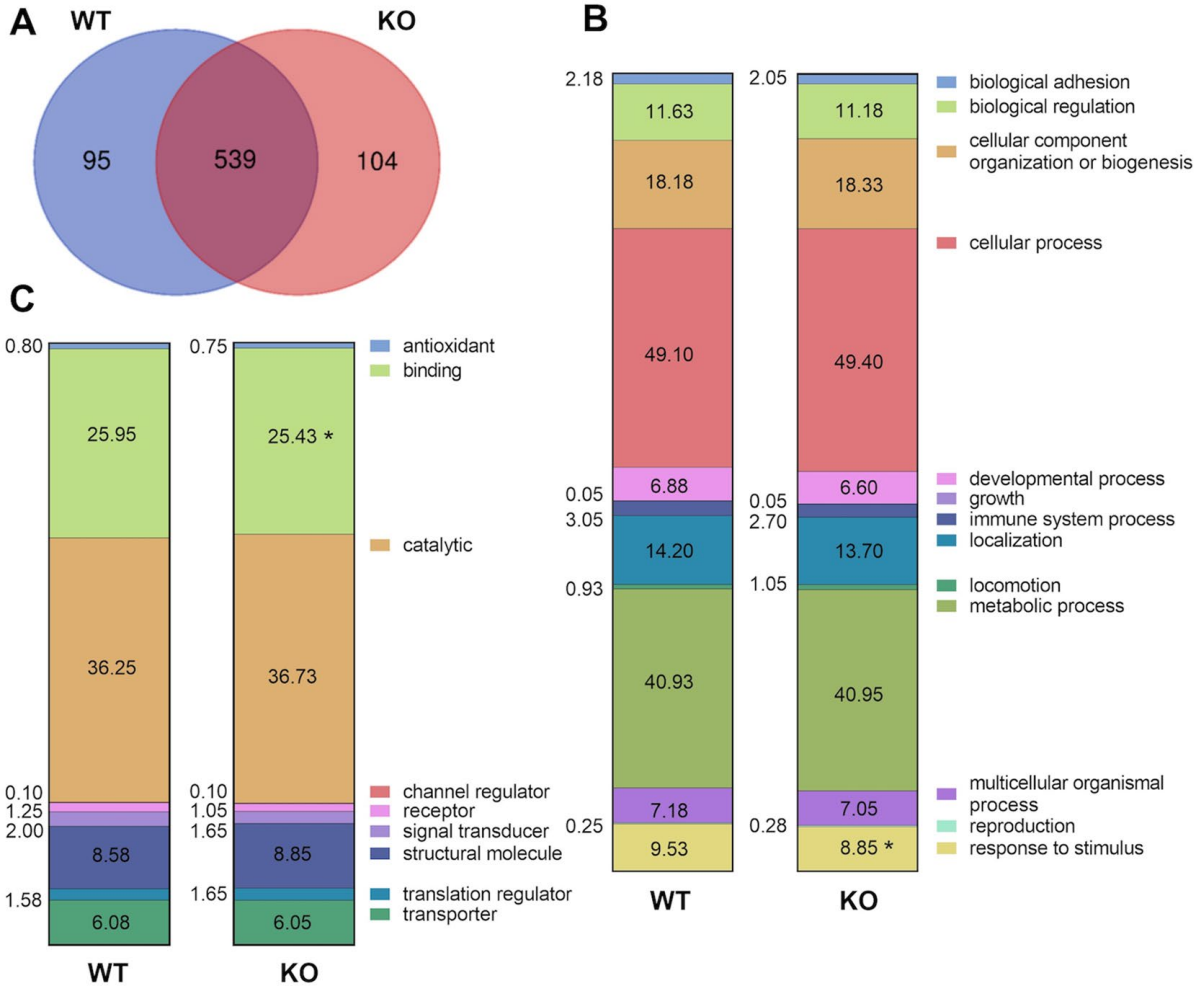
LC/MS–MS analysis in decellularized subcutaneous adipose tissue from WT and KO mice. (**A**) Venn diagram summary showing protein variability between WT and KO (only included proteins that were expressed in all replicates). Comparison between (**B**) biological processes, and (**C**) molecular functions signaling pathways showed that WT and KO ECM had differences in the “binding” and “response to stimulus” signaling pathways. Proteins were quantified using the exponentially modified protein abundance index (emPAI), which relates the number of unique peptides observed for a specific protein to the number of observable peptides in the sample. n = 3 biological samples with 3 technical replicates. The significance threshold was set at *p* < 0.05, with a False Discovery Rate of 5% using ANOVA.

Mice absent in laminin α4 (KO) are resistant to obesity and exhibit increased beiging of SubQ adipose tissue, increased energy expenditure, and enhanced insulin sensitivity^18^. LC/MS–MS analysis was used to evaluate ECM composition of the SubQ adipose tissue from WT and KO mice. The total proteins in mice were evaluated and grouped according to presence in WT and KO mice (Fig. 1A) and then categorized based on general biological processes (Fig. 1B) or signaling pathways (Fig. 1C) that the proteins belonged to. While there was significant overlap in proteins present in adipose tissue from the two WT and KO mice, specific differences were identified with regards to proteins involved in “binding” processes and “response to stimulus” signaling pathways.

**Figure 2:**
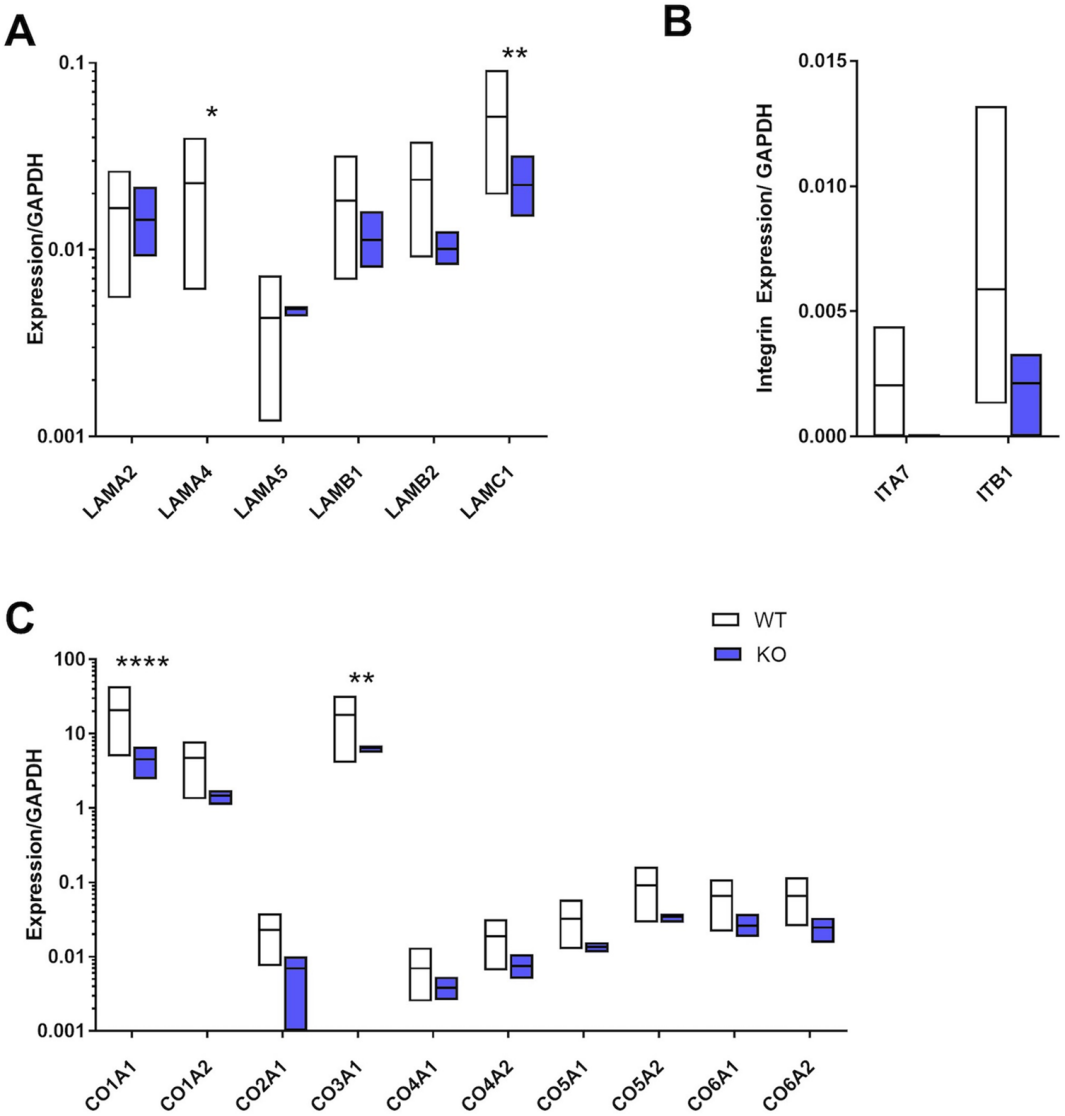
Matrix protein and integrin differences between subcutaneous adipose tissue from WT and KO mice. (**A**) In addition to the expected absence of laminin α4 in the adipose ECM, there was a significant decrease in laminin gamma 1 (LAMC1). (**B**) When evaluating proteins involved in cell binding and integrin signaling, integrins α7 (ITA7) and β1 (ITB1) were dramatically reduced in the subcutaneous adipose from KO mice. (**C**) Both collagen 1A1 (CO1A1) and collagen 3A1 (CO3A1) were significantly lower in subcutaneous adipose tissue from KO mice. n = 3 biological samples with 3 technical replicates. Data are presented as mean ± standard error of the mean with *p < 0.05, **p < 0.01, ****p < 0.0001 as significant from one-way ANOVA.

Proteins in the "binding" pathway were examined and broad differences in the ECM profile between adipose from WT and KO mice were identified. In addition to the expected absence of LAMA4 (*p* < 0.05), there was significantly lower levels of laminin gamma 1 (LAMC1) (*p* < 0.01, Fig. 2A). Nearly all collagen molecules identified were lower in SubQ adipose tissue from KO mice, with both collagen 1A1 (CO1A1) and collagen 3A1 (CO3A1) showing significantly different levels (*p* < 0.0001 and *p* < 0.01 respectively, Fig. 2C). When evaluating proteins involved in cell binding and signaling, integrins α7 (ITA7) and β1 (ITB1) were found to be lower in the KO mice (Fig. 2B).

### Expression of beige markers varies with extracellular matrix substrate

**Figure 3:**
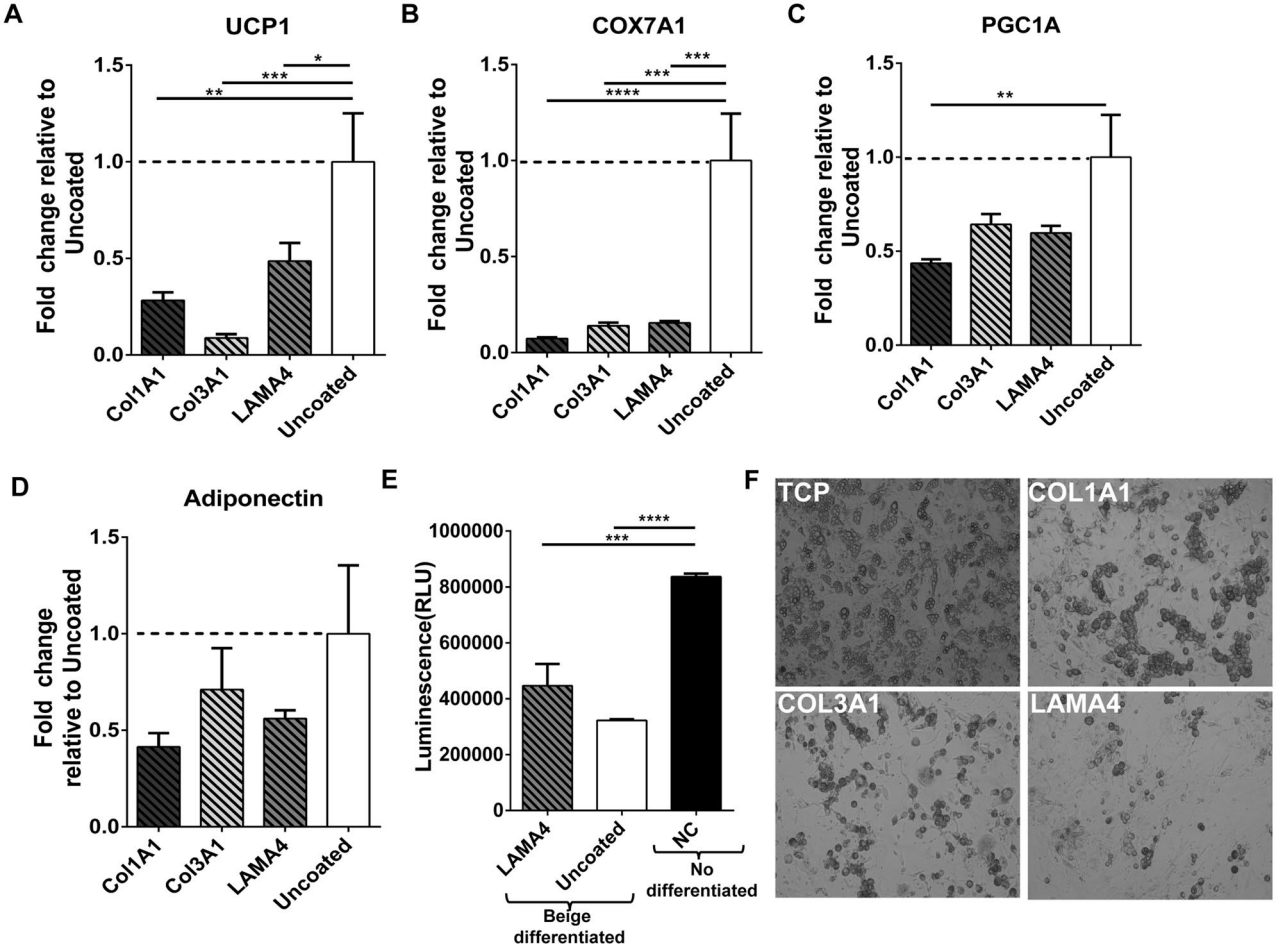
Matrix proteins effect in ASCs beige differentiation. Fold change of (**A**) UCP1, (**B**) COX7A1, (**C**) PGC1A and (**D**) adiponectin expression by ASCs cultured and differentiated on CO1A1, CO3A1 or LAMA4 surfaces, relative to uncoated tissue culture plates. UCP1 and other beige markers (COX7A1and PGC1A) expression is decreased in cells cultured on LAMA4, CO1A1 and CO3A1 surfaces, while adiponectin levels are unchanged. n = 3 technical replicates per sample. Data are presented as mean ± standard error of the mean with *p < 0.05, **p < 0.01, ***p < 0.001 as significant from one-way ANOVA followed by all pairwise multiple-comparison procedures (Dunn method). (**E**) Luminescence levels (proportional to H2O2 levels) measured by the ROS-GLO assay in cells differentiated in LAMA4 coated surfaces, uncoated surfaces or in undifferentiated cells as negative control (NC). Data represent mean ± standard error from n = 4 replicates per sample. ***p < 0.001, ****p < 0.0001 compared to control groups, as analyzed with ANOVA.

Based on the results obtained from MS analysis, adipose derived stem cells (ASCs) isolated from WT SubQ WAT were cultured on surfaces coated with LAMA4, CO3A1 and CO1A1 and exposed to a beige differentiation protocol. Cells were able to proliferate and differentiate on all surfaces. However, UCP1 expression was lower with cells cultured on LAMA4, CO3A1 and CO1A1 surfaces relative to controls (Fig. 3A). Specifically, cells differentiated on a LAMA4 surface had a 51.4% reduction in UCP1, while cells differentiated on CO3A1 and CO1A1exhibited a 91.16% and 71.7% reduction relative to uncoated surfaces, respectively. The expression of additional beige markers cytochrome c oxidase subunit isoform COX7A1 (Fig. 3B) and peroxisome proliferator-activated receptor-γ coactivator-1α1 (PGC1A) (Fig. 3C) was also analyzed. Similar to UCP1, there was a significant decrease in COX7A1 in differentiated cells on CO3A1, CO1A1 and LAMA4. PGC1A levels were also lower in cells on the coated surfaces, with a significant decrease on CO1A1 compared to uncoated surfaces. These results demonstrate that culture of ASCs on the ECM proteins present at higher levels in WT adipose tissue results in lower UCP1 expression. Adiponectin levels were analyzed as a general measures of adipogenic differentiation of the ASCs. Results showed lower expression levels of adiponectin on the ECM molecules but the differences were not significant (Fig. 3D). The levels of reactive oxygen species (ROS) in culture were also examined in culture on the surfaces (Fig. 3E). UCP1 has been shown to reduce ROS production^25^. ROS levels were highest in undifferentiated cells that do not express UCP-1. ROS levels were lower in differentiated cells on LAMA4 and uncoated surfaces consistent with increased UCP1 expression. However, cells on LAMA4 coated surfaces had higher ROS levels compared to uncoated surface correlating with the lower UCP1 production on these surfaces.

### Extracellular matrix proteins and integrin levels vary with adipose depot

**Figure 4:**
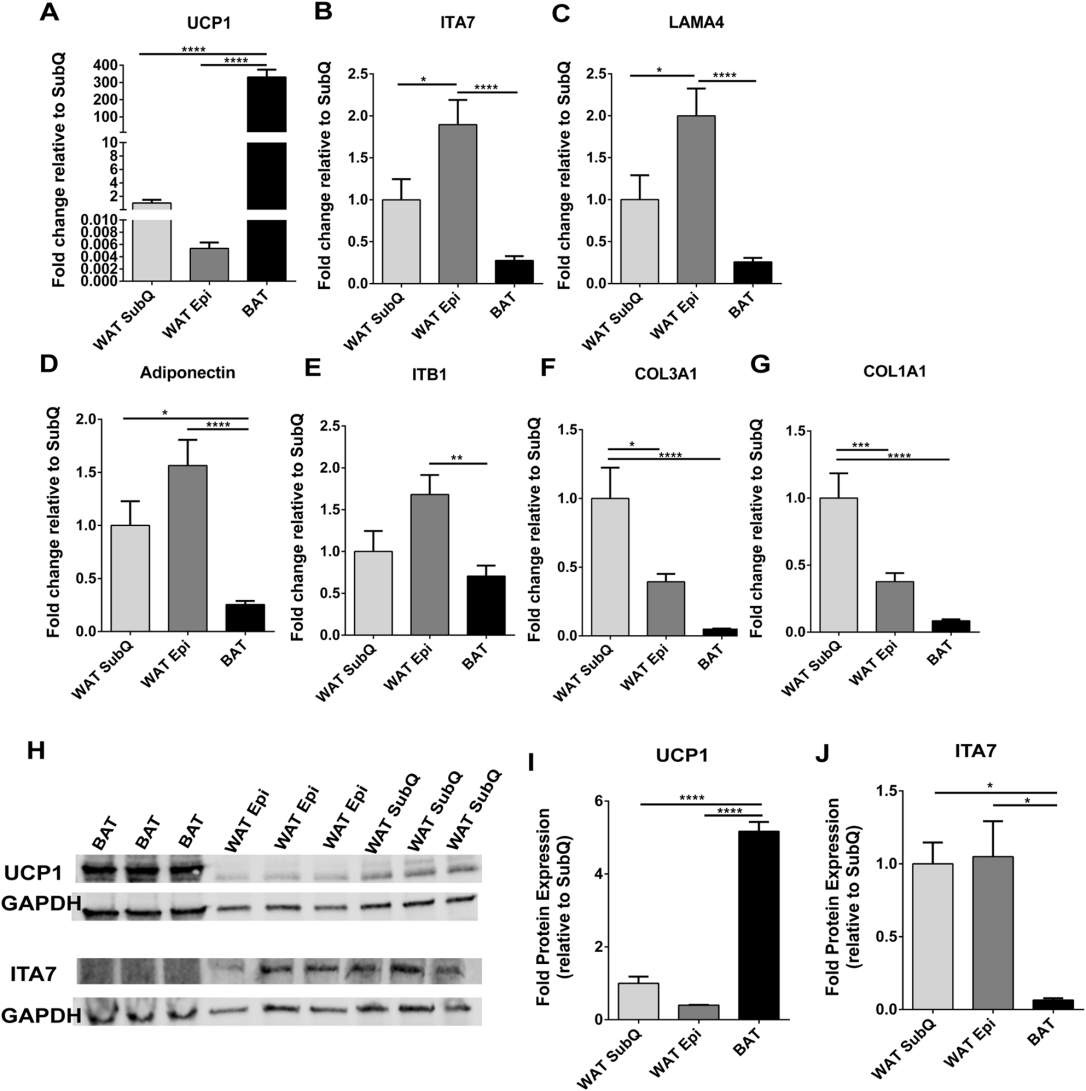
ITA7 and LAMA4 are inversely correlated with the expression of UCP1 in adipose tissue. (**A**) UCP1, (**B**) ITA7, (**C**) LAMA4, (**D**) Adiponectin, (**E**) ITB1, (**F**) CO3A1 and (**G**) CO1A1 fold gene expression in epidydimal white adipose tissue (WAT Epi) and brown adipose tissue (BAT) with respect to subcutaneous white adipose tissue (WAT SubQ). n = 6 animals with two replicates per adipose tissue sample. (**H**) Protein expression of UCP1 and ITA7 in different adipose depots was assessed by western blot. Full-length blots are presented in Supplementary Fig. 5. (**I**) UCP1 and (**J**) ITA7 band intensity was quantifed and normalized to total GAPDH signals. n = 3 animals. Data are presented as mean ± standard error of the mean with *p < 0.05, **p < 0.01, ****p < 0.0001 as significant from one-way ANOVA followed with Tukey's multiple comparisons test.

The expression of UCP1 varies with adipose depot. BAT and two different kinds of WAT, SubQ and Epi, were isolated from WT mice, and the gene expression levels of of ITA7, ITB1, CO1A1, LAMA4, and CO3A1 (Fig. 4) were examined by RT-PCR. As expected, UCP1 mRNA levels were significantly higher in BAT compared to both types of WAT (Fig. 4A). While UCP1 expression levels were higher in SubQ the difference with Epi was not significant. Adiponectin gene levels were lower in BAT compared to both Epi (*p* < 0.0001), and SubQ WAT (0.01), with no difference among the WAT depots (*p* = 0.09) (Fig. 4D). Both ITA7 and ITB1 were significantly lower in BAT. ITA7 mRNA levels in BAT were 84% lower than Epi (*p* < 0.0001) and 66% than SubQ (*p* = 0.068). Interestingly, ITA7 levels were significantly higher in Epi when compared to SubQ (*p* = 0.016) (Fig. 4B). ITB1 levels were significantly lower in BAT compared to Epi (58% *p* < 0.001), and lower but not statistically significant compared to SubQ (*p* = 0.059, Fig. 4E).

The ECM proteins LAMA4, CO1A1 and CO3A1 were also lower in BAT . LAMA4 levels were significantly lower in comparison to Epi (*p* < 0.0001) and lower than SubQ WAT (*p* = 0.1). LAMA4 levels in Epi were significantly higher than SubQ WAT (*p* = 0.018) (Fig. 4C). Additionally, CO1A1 and CO3A1 levels were lower in BAT compared to SubQ (*p* < 0.0001), and higher in SubQ compared to Epi (*p* = 0.0076 in CO1A1 and *p* = 0.0002 in CO3A1; Fig. 4F,G).

UCP1 protein levels were analyzed by western blot and results confirmed RT-PCR results, with higher UCP1 levels in BAT compared to other adipose depots but no difference between SubQ and Epi (Fig. 4H-I). Protein levels of ITA7 were also greater in both Epi and SubQ WAT compared to BAT (Fig. 4J). A broad observation is that UCP1 levels inversely correlate with LAMA4, ITA7, and ITB1 in adipose tissues.

### Integrin α7 protein expression in subcutaneous adipose tissue

**Figure 5:**
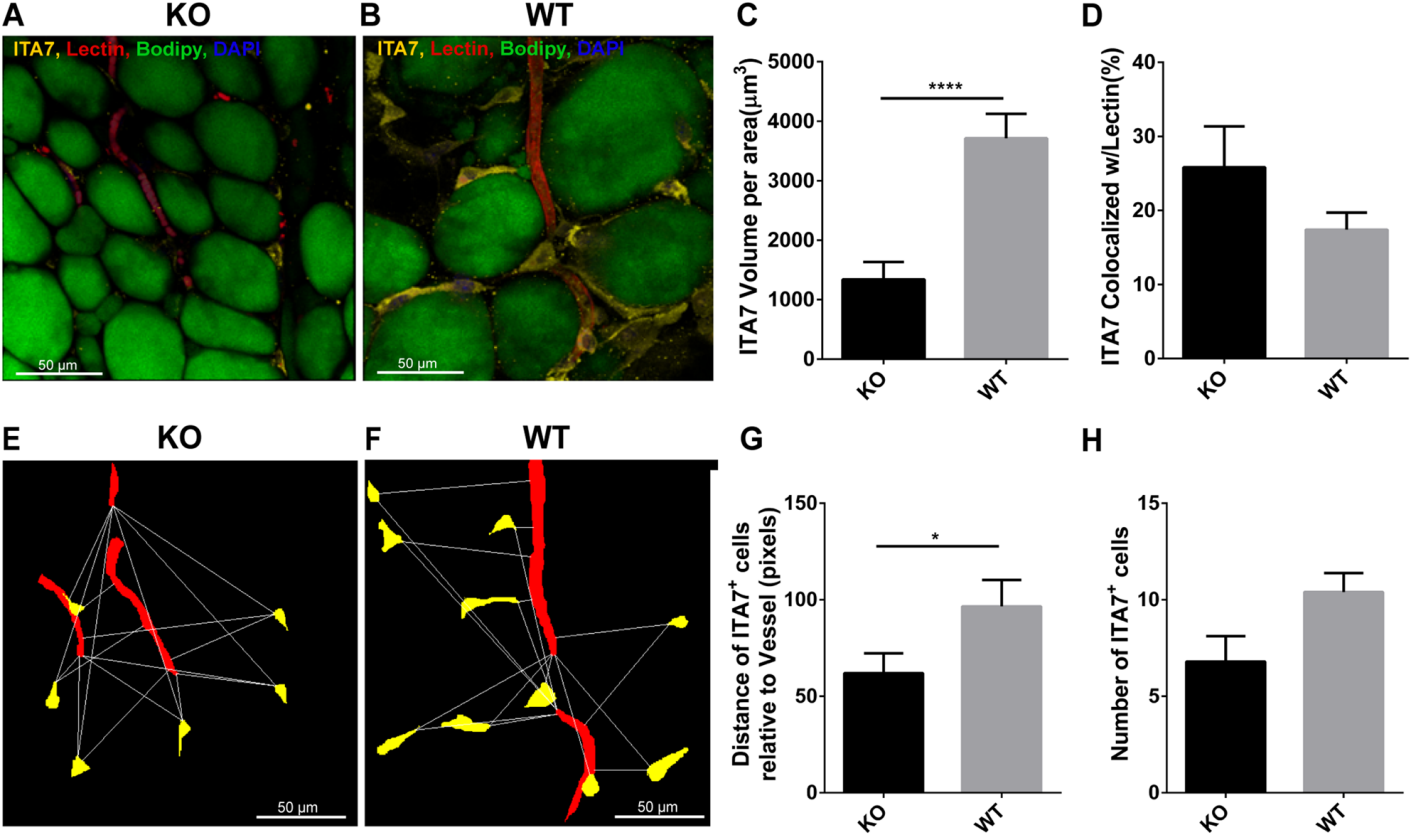
Analysis of ITA7 levels and spatial distribution in KO and WT adipose tissue. Immunohistochemical confocal images of fixed adipose tissue from (**A**) KO mice and (**B**) WT mice. Formaldehyde-fixed tissues were stained with antibodies against ITA7. Endothelial cells were counterstained with isolectin (red), the fatty acid with BODIPY (green) and the nucleus with DAPI (blue). (**C**) ITA7 volume was 64% lower in KO tissue compared to WT (1338 ± 296 μm^3^ in KO vs. 37,013 ± 409 μm^3^ in WT, *p* < 0.0001). (**D**) There is not significant difference in the percent of ITA7 colocalized with endothelial cells between KO and WT adipose tissue (*p* = 0.17). Values are mean ± standard error from unpaired t test with Welch's correction. Two tailed p value was calculated. n = 30 images from 4 WT animals and 25 images from 3 KO animals. (**E**,**F**) Representative masks of the images used to quantify the spatial proximity of ITA7^+^ cells with vessels of KO and WT adipose tissue. cytoNet analysis showed that (**G**) ITA7^+^ cells were closer to vessels in KO tissue compared to WT tissue (61.9 ± 14.6 pixels in KO vs. 96.5 ± 11.8 pixels in WT, *p* = 0.046), and (**H**) the number of ITA7^+^ cells was greater in WT tissue compared to KO, although this difference did not reach significance (6.8 ± 1.1 cells in KO vs 10.4 ± 1.1 cells in WT, *p* = 0.059).

This is the first study to identify a potentially important role for ITA7 in adipose tissues. A confocal imaging and staining protocol was used to investigate the distribution of ITA7 positive cells in adipose tissue. Adipocytes, vasculature, and ITA7 were simultaneously stained in adipose tissue from KO and WT mice (Figs. 5A,B). The volume of ITA7 was 64% lower in SubQ adipose tissue of KO mice compared to WT (1338 ± 296 μm^3^ in KO vs. 3713 ± 409 μm^3^ in WT, *p* < 0.0001) (Fig. 5C). Adipose precursor cells generally take on a perivascular phenotype^26^. In examining both KO and WT adipose tissue, ITA7 staining was observed in cells present in the stromal tissue (ITA7^+^ cells) as well as lining the vasculature. Quantitative, the colocalization between ITA7 and vessels (lectin) was calculated. 26 ± 5% of ITA7 was colocalized with lectin in KO mice and 17 ± 2% of ITA7 was colocalized with lectin in WT (Fig. 5D). Although greater ITA7 and lectin colocalization was observed in KO tissue, the difference did not reach our criteria for significance (*p* < 0.05).

To get a further analysis on the tissue distribution of ITA7^+^ cells, we used cytoNet which is a robust method to quantify the spatial organization of cell communities. The analysis of spatial proximity of ITA7^+^ cells with both adipocytes and vessels was performed by evaluating the proximity of ITA7^+^ cells in relation to a threshold distance (Figs. 5E,F). Results showed that ITA7^+^ cells in KO tissue were on average closer to vessels than ITA7^+^ cells in WT tissue (*p* = 0.046). The number of ITA7 positive cells was also quantified using cytoNet. There was a greater number of ITA7^+^ cells in WT tissue compared to KO, although this difference was not significant (*p* = 0.059), consistent with both MS analysis and our standard analysis of the stains.

Adipose tissues from KO and WT mice were stained for BODIPY (adipocytes), CD68 (a macrophage marker) and ITA7 in order to gain further insight into the types of cells expressing ITA7 (Supplemental Fig. 1). Quantitatively, the amount of CD68 cells was significantly lower in SubQ adipose tissue of KO mice compared to WT (501 ± 1305 μm^3^ in KO vs. 4034 ± 697 μm^3^ in WT, *p* = 0.0029) (Supplemental Fig. 2) suggesting a decrease of macrophages in KO animals. Interestingly, the colocalization analysis with ITA7 showed that the majority of CD68 are also positive for ITA7 with no significant difference between KO and WT adipose tissue (83 ± 5% of CD68 cells in KO vs. 72 ± 4% in WT, *p* = 0.07). From the overall ITA7 volume, 16 ± 5% in KO and 26 ± 4% in WT were CD68 cells. The majority of the ITA7 positive regions lined differentiated adipocytes. These results suggest that ITA7 is expressed in both macrophages and adipocytes.

In addition to the ITA7 analysis, we also analyzed other tissue characteristics including adipocyte size and vascular density. Mean adipocyte diameter was significantly smaller in KO SubQ adipose compared to WT (50.6 ± 1 μm in KO vs 55.9 ± 1.1 μm in WT, *p* = 0.0004 F_(1,547) _= 12.7; n = 550 adipocytes from 7 animals). Vascular parameters were not different between the two conditions. Vascular diameter was 4.7 ± 0.14 μm in WT, and 5.0 ± 0.15 μm in KO (*p* = 0.12, F_(1,348)_ = 2.3; n = 350 vascular diameters from 7 animals). Vascular volume was 1164 ± 291.5 μm^2^/area for KO and 1613 ± 243.9 μm^2^/area for WT (F_(1,49)_ = 0.018, *p* = 0.89; n = 50 images from 7 animals). These results were further confirmed by cytoNet analysis. The number of vessels was not different in WT and KO images. The average number of vessels per image was 4 ± 0.5 in KO and 3.4 ± 0.5 in WT (*p* = 0.45, n = 5 images per condition).

### Integrin α7 and Laminin α4 decrease during beige differentiation

**Figure 6:**
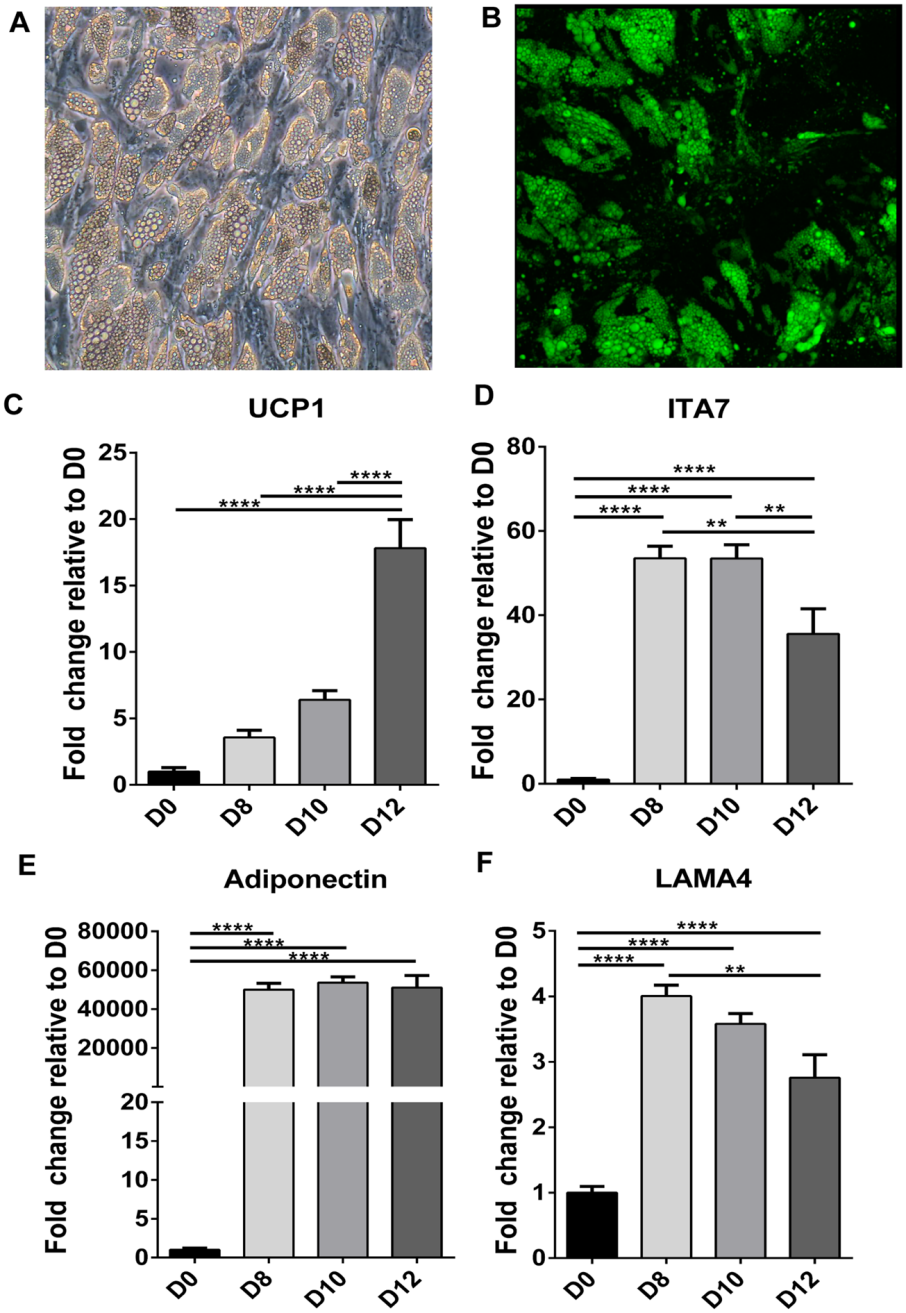
Timeline expression of UCP1, ITA7, Lama4 and adiponectin mRNA levels during beige differentiation of hASCs. (**A**) Representative phase microscopy picture and (**B**) fluorescent image counterstained with BODIPY, of hASCs at 12 days of differentiation. mRNAs levels of (**C**) UCP1, (**D**) ITA7, (**E**) adiponectin and (**F**) LAMA4 were evaluated in hASCs relative to D0 (undifferentiated cells) at 8 (D8), 10 (D10) and 12 (D12) days of beige differentiation.UCP1 increases with time with a significant increase at D12. At D12 ITA7 and LAMA4 had a significant decrease compared with previous days (D8 and D10). Adiponectin significantly increases between D0 and D8. Values are means ± standard errors, from one-way ANOVA analysis followed with Tukey's multiple comparisons test. **p < 0.01; ****p < 0.0001; n = 2 different experiments with 4 replicates per group per experiment.

Human adipose derived stem cells (hASCs) were next induced to form beige adipocytes and ITA7, LAMA4 and UCP1 levels were evaluated during the time course of differentiation. Phase microscopy and immunofluorescence confirm the morphological changes of the cells when differentiated and the lipid loading that occurs (Fig. 6A,B). UCP1 levels gradually increased over the 12 days of differentiation with the greatest increase from 10 to 12 days (Fig. 6C). ITA7 and LAMA4 levels showed a significant increase from day 0 to day 8 over the time course where there was not a significant increase in UCP1 expression differentiation (Figs. 6D,F). However, from day 8 to day 12 LAMA4 and ITA7 levels showed an inverse relationship with UCP1 with a significant decrease level of expression from day 10 to day 12 simultaneous with the dramatic increase in UCP1. At 8 days, a major increase in adiponectin also occurs, with no further significant changes over the course of differentiation (Fig. 6E). Similarly, other beige markers such as PGC1A, COX7A1, cell death activator CIDE-A (CIDEA) and iodothyronine deiodinase 2 (DIO2) showed a significant increase between d0 and d8 with no further increase between d8 and d12 (Supplemental Fig. 3).

### Knocking down integrin α7 leads to increased expression of UCP1

**Figure 7:**
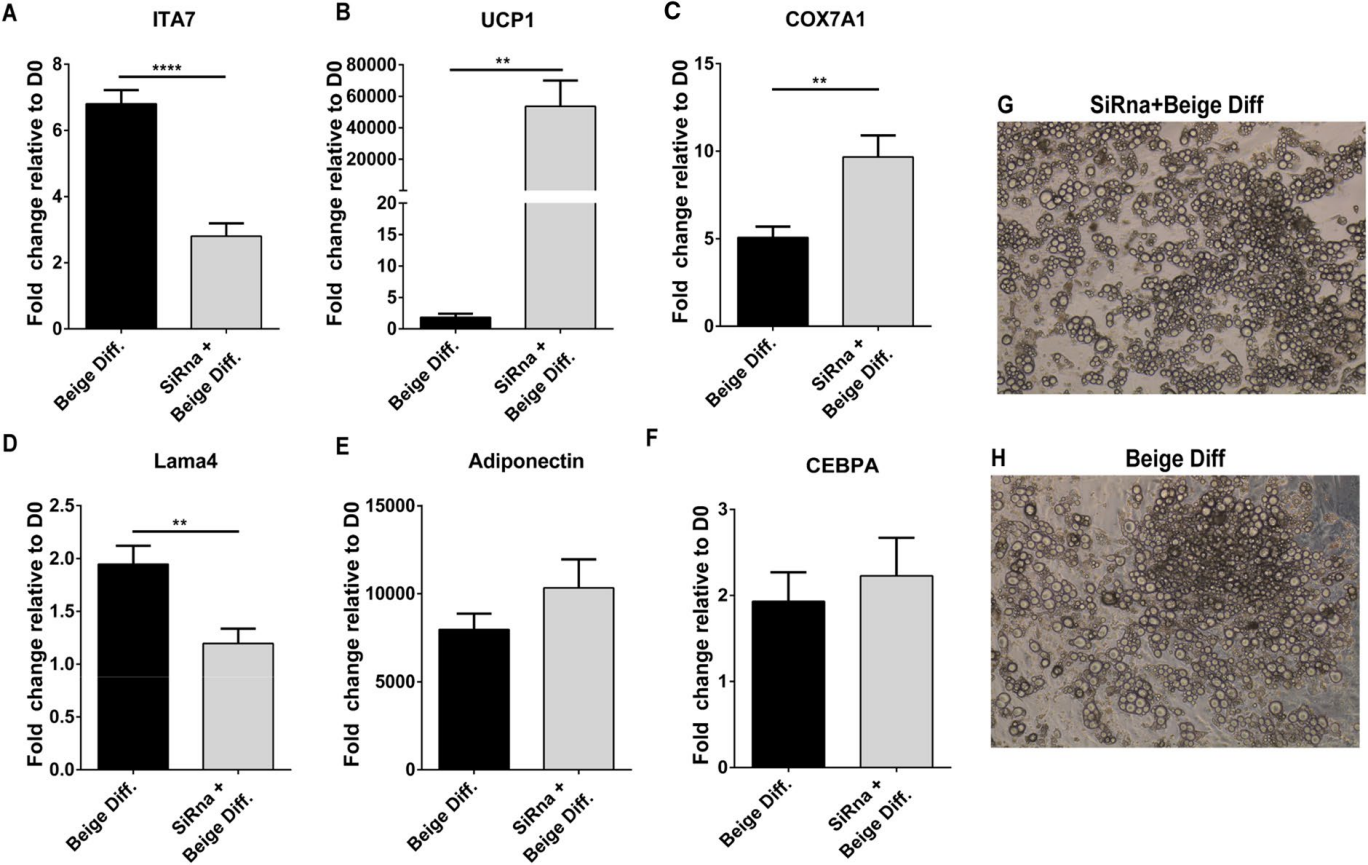
Effect of ITA7 knockdown on beige adipocyte differentiation and LAMA4 expression. (**A**) Efficacy of ITA7 siRNA in knocking down levels of ITA7 in ASC differentiated to beige adipocytes (****p < 0.0001). (**B**) UCP1 (**p = 0.0055), (**C**) COX7A1(**p < 0.01), (**D**) LAMA4 (**p = 0.0021), (**E**) adiponectin and (**F**) CEBPA expression in ITA7 knockdown cells. n = 3 experiments with 3 replicates per group per experiment. (**G**–**H**) Representative brightfield images showing lipid loading of the adipocytes with or without ITA7 knockdown. Values are means ± standard errors, from unpaired t test with Welch's correction. Two tailed p value was calculated.

Based on these data integrin α7 serves as a potential target for therapeutic intervention. To evaluate ITA7 regulation as a means for increasing beige adipose formation, ASCs were transfected with ITA7 siRNA during adipogenic differentiation. In ASCs transfected with ITA7 siRNA, ITA7 gene levels were reduced by 59% (from 6.805 ± 0.41 to 2.806 ± 0.38; Fig. 7A. These ASCs also exhibited a substantial increase in UCP1 (Fig. 7B) and COX7A1 (Fig. 7C) expression. Interestingly, LAMA4 was also downregulated (Fig. 7D) in the cells transfected with ITA7 siRNA. Adiponectin and C/EBPalpha expression was not different between groups (Fig. 7E-F) suggesting that basic adipogenesis of the cells was not affected. Indeed, brightfield images of differentiatiated adipocytes show lipid loading in cells with or without ITA7 knockdown (Fig. 7G-H).

## Discussion

In this study extracellular matrix-cell interactions that play a role in beige adipocyte differentiation were identified. Laminin α4 KO mice are resistant to obesity, exhibit enhanced energy expenditure, and increased beige adipose formation^18^. Broad changes in adipose ECM composition was observed in mice. The overall ECM composition is altered in complex ways, including a reduction in a number of collagen isoforms in addition to LAMA4. In addition, ITA7 was also lower in comparison to adipose from WT mice with levels appearing to correlate with LAMA4 level in multiple tests. The results of these studies provide a new list of potential targets for treatment of obesity or metabolic disease.

The basement membrane of adipocytes consists primarily of various laminin isoforms and collagen^27^. These components provide attachment points for integrins and other extracellular matrix receptors, such as CD36 and CD44; anchored in the adipocyte membrane^28^. The importance of the matrix interaction with adipogenesis has been previously demonstrated. For instance, blocking collagen synthesis in ASCs inhibits differentiation; and collagen VI is sufficient to restore the adipogenic potential^22^. Results from the current study provide insight into the differences between ECM in beige and white adipose tissue by looking into ECM protein changes in the SubQ adipose tissue from WT and KO mice. LC/MS analysis revealed changes in the ECM profile. CO1A1 and CO3A1 were at significantly lower levels in KO mice compared to WT. Additionally, CO3A1 levels were lower in BAT compared to WAT suggesting that thermogenic adipose tissue (brown and beige) have lower levels of CO3A1 and CO1A1. Collagen deposition makes the ECM less flexible which is something that occurs during fibrosis, the hallmark of obesity and type 2 diabetes^29,30^. CO1A1 and CO3A1 are indeed two markers of fibrosis and it has been shown that they play a role in fibro‐adipogenic precursor differentiation and fat deposition^31^. Higher levels of CO1A1 and CO3A1 were associated with higher levels of fibro-adipogenic progenitors in muscle as well as greater adipose tissue deposition. The decrease in ECM proteins that we observed in our results, specifically in CO1A1 and CO3A1 can alter the flexibility of the tissue suggesting that a tissue more flexible favors the development of thermogenic adipose tissue. Future studies will look into how stiffness and mechanical properties impact the process of beiging white adipose tissue.

ASC plated on CO1A1, CO3A1 or LAMA4 surfaces and induced to differentiate into beige adipocytes, exhibited lower levels of UCP1 compared to cells differentiated on uncoated tissue culture plates. These differences suggest that a coordinated change in ECM proteins may occur during the beiging of WAT and provides multiple potential targets when developing therapeutic approaches. The ECM composition in adipose tissue changes during development and is different between adipose depots^22,32,33^. For instance, SubQ WAT of rats express higher levels of type I and III collagens compared to visceral WAT^33^. Our results showed that CO3A1 is indeed greater in SubQ adipose tissue compared to visceral adipose tissue and BAT. Lowering levels of CO3A1 in SubQ adipose tissue may create an environment that helps promoting differentiation of stem cells into beige adipocytes.

ECM proteins are often linked to the cytoskeleton through integrins which are a class of transmembrane proteins. Integrins are heterodimers of alpha and beta subunits, the combination of each dictates ligand specificity^34^. Integrin interactions with ECM molecules can activate signaling pathways that influence gene expression and cell function. Classification analysis of the protein results suggest that cell binding and integrin signaling are reduced in the KO beige adipose tissue. Specifically, ITA7 and ITB1 were reduced in the KO beige adipose tissues. To confirm these results we evaluated the levels of ITA7 and ITB1 in WAT and BAT of WT mice. The levels of both integrins were lower in BAT compared to WAT. These results indicate that low levels of these integrins are characteristic of the ECM of thermogenic adipose tissue. Therefore, lowering levels of these integrins in WAT may induce beiging of the adipose tissue. ITB1 plays a role in mediating cellular interactions with many ECM proteins^35^. Also, ITB1 is upregulated during hypertrophic growth, as is the activity of downstream effector kinases^36^. ITB1 is widely present in many cells, playing a role in a broad range of cellular processes, making ITB1 a difficult target for therapeutic purposes. ITA7 has been linked with the regulation of cell adhesion and migration^37^, and although its role in adipocytes has not been studied, ITA7 is strongly upregulated in differentiated white adipocytes compared to ASC^38,39^. Our results showed that ITA7 levels in hASCs are also upregulated in the initial stages of beige adipocyte differentiation, but at later stages it decreases as UCP1 levels increase. These results suggest that ITA7 upregulation is needed to start the differentiation process and might induce changes in the shape of adipocytes, and facilitate their localization at laminin-rich sites as it does in other cells such as myoblasts^40–42^. At later stages, our results demonstrates that differentiated beige adipocytes present lower levels of ITA7. In contrast, further upregulation of ITA7 have been seen in insulin resistant and hypertrophied 3T3-l1 adipocytes^38^.

Recent studies have shown that brown/beige adipocytes can also be derived from muscle progenitor cells. Muscle progenitor cells can be differentiated, in part, by their ITA7 expression. Progenitor cells negative for ITA7 have a significantly higher propensity for UCP1 expression consistent with results we have seen here^43–46^. To confirm the implication of ITA7 in modulating the metabolic function of adipocytes, we knocked down the expression of ITA7 in ASC and induce them to beige differentiation. In agreement with our other results, ITA7 knockdown is able to enhance UCP1 expression making it a possible target to induce beige adipose tissue.

Staining for ITA7 in SubQ adipose tissue, not only confirms that ITA7 is lower in KO mice but also provides insight into the potential function of these cells. Cells positive for ITA7 were present both in the stroma and perivascularly, possibly suggesting that ITA7 stains multiple cell types, precursor cells in the perivascular space and other cells types in the stroma. Indeed, ITA7 stained the majority of CD68 cells in the stroma indicating that a subset of the ITA7 positive cells are macrophages. Macrophages have been shown to play a role in adipogenesis of fibroadipogenic progenitors^47^, but it remains unknown the role that immune cells have on beige adipocytes. CD68 volume was lower in KO tissue, in agreement with previous studies showing that CD68 tend to increase with BMI and to decrease with agonists for peroxisome proliferator–activated receptor- (PPAR-)^48^. ITA7 present in the adipose tissue from KO mice exhibited a different distribution than in tissue from WT animals. It is important to understand the cell spatial distribution since it has been demonstrated that there is a close spatial and temporal interrelationships between blood vessel formation, adipogenesis and stromal cells^49^. In KO adipose the ITA7^+^ cells in the stroma were closer to the vessels on average. Although it the reason for this difference in ITA7 spatial distribution is unclear, it may result, in part, from the difference in size of adipocytes in WT and KO adipose tissue. The increase in beige adipose tissue means that the adipocytes are smaller in KO mice which can reduce the effective distance from vessels. The role of the ITA7 spatial distribution in beige adipogenesis remains to be studied. More ITA7 positive cells could indicate an increase in ASC differentiation to white adipocytes, since ITA7 increases during the process of white adipocyte differentiation^38,39^. Less hyperplasia (increased in adipocyte number) in SubQ of KO mice could explain their resistant to obesity and enhanced metabolic function that was previously observed^18^. Therefore, lowering ITA7 in tissue might switch the differentiation of stromal cells from white to beige adipocytes.

All of our results indicate a direct correlation between LAMA4 expression and ITA7. ITA7 specifically binds to laminin, the substrate that is an important ECM component of adipocytes containing large fat vacuoles^50,51^. A study by Morandi et al.^39^ performed gene profiling of 18 alpha integrins in ASCs and differentiated adipocytes, and suggested that ITA7 is the responsible for laminin-dependent signaling in differentiating ASCs. In our study we demonstrated that ITA7 levels regulates beige adipocyte differentiation and downregulation of ITA7 occurs in LAMA4 KO adipose tissue. Therefore, ITA7 could be responsible for LAMA4-dependent signaling in beige differentiating ASCs. Future studies will look into the Notch signaling pathway as a mechanism of LAMA4 regulation of UCP1 expression. In the absence of LAMA4 the Notch pathway is supressed^52^ and inhibition of Notch signaling has been shown to result in browning of white adipocytes^53^.

### Conclusion and future perspective

The results of this study provide unique insights into adipose function by investigating changes in extracellular matrix components that alter thermogenic capacity of adipocytes. Specifically, we found that ITA7 modulates the metabolic function of the adipocytes. In addition, the ECM of thermogenic adipose tissue is characterized to have lower levels of CO1A1 and CO3A1 compared to WAT. This information identify new therapeutic targets for obesity and metabolic diseases. A better understanding of the underlying causes of these characteristics of brown and beige fat allow us to specifically manipulate these cells to improve systemic energy metabolism and glucose homeostasis.

## Methods

### Animal models

The development and characterization of laminin α4 knockout mice (KO) has been described previously^12^. Wild type (WT) mice used are C57 BL/6 mice (Charles River) as the KO mice were generated on this background. For the studies described here the mice were fed a standard chow diet ad libitum. All animal procedures were approved by the IACUC at the University of Chicago.

### Tissue harvest

Mice at 13–15 weeks of age were sacrificed and various adipose tissue depots harvested from both WT and KO mice. WAT was isolated from subcutaneous (SubQ) and epididymal (Epi) depots, and BAT was isolated from the interscapular region. The number of mice used per experiment are provided in each section below.

### Mass spectrometry

Liquid chromatography/tandem mass spectrometry (LC/MS) was used to identify the ECM components and the relative quantities in WAT from WT and KO mice. SubQ adipose tissue was frozen in liquid nitrogen. After mincing, the tissue was suspended in CHAPs buffer in PBS and stirred in a hot plate until dissolved, followed by incubation overnight at 37 °C with 135 RPM. The buffer was removed and tissue suspended in Tris/EDTA buffer prior to freezing at -80 °C. To remove lipids, the tissue was thawed and incubated in isopropanol for 48 h. The adipose tissue samples were solubilized in Rapigest (Waters Milford, MA) containing 50 mM ammonium bicarbonate, and sonicated at 2 × 15 s with 0.5 s pulsing. DTT and IAN were added to reduce and alkylate, respectively, the proteins prior to Lys-C/Trypsin digestion for 45 min and then overnight. Samples were then acidified with TFA to remove Rapigest, and protein/peptide concentration was determined via amino acid analysis prior to injecting samples into the mass spectrometer. LC/MS–MS analysis was performed on a Thermo Scientific Orbitrap Elite mass spectrometer coupled to a Waters nanoACQUITY UPLC system, using a Waters Symmetry® C18 180 μm 9 20 mm trap column and a 1.7 lm, 75 μm × 250 mm nanoAcquity™ UPLC™ column (38 °C) for peptide separation across a 140 min run. MS was acquired in the Orbitrap (300–2000 m/z) using 1 microscan, a full max ion time of 500 ms, and a resolution of 30 000. MS–MS was acquired in the Ion Trap using collision-induced dissociation (CID) for up to 15 MS–MS analyses per MS scan. Minimum signal required was 500, dynamic exclusion was set to 60 s, and the normalized collision energy was 35. Mascot Distiller was used to generate peak lists, and the Mascot search algorithm was used for searching against the Swiss Protein database with and without taxonomy restricted to human. Carbamidomethyl Cys, citrullination of Arg, oxidation of Met, Pro, and Tyr, and Nitrosylation of Cys were entered as variable modifications. Two missed tryptic cleavages were allowed, precursor mass tolerance was set to 10 ppm, and fragment mass tolerance was set to 0.2 Da. The significance threshold was set at *p* < 0.05, with a False Discovery Rate (FDR) of 5%. Three biological samples were run, with 3 technical replicates for each sample. Proteins were quantified using the exponentially modified protein abundance index (emPAI), which relates the number of unique peptides observed for a specific protein to the number of observable peptides in the sample^54^.

### Immunohistochemistry

SubQ fat was harvested from animals using sterile techniques, cut into small pieces and fixed in 4% paraformaldehyde for 24 h at 4 degrees. Tissue pieces were then washed with phosphate buffered saline (PBS), permeabilized with 0.5% triton in PBS and blocked with 10% donkey serum; followed by incubation with either 1) ITA7 antibody (1:100, Novus Biologics NBP1-86,118) and *Griffonia simplicifolia* isolectin conjugated with Rhodamine to labels endothelial cells, or 2) ITA7 antibody and CD68 antibody (1:100, Santa Cruz sc-70761) to label macrophages for 48 h at 4 degrees. After washes (3 × 15 min), the tissues were incubated with second antibodies (Alexa Fluor 647 Donkey Anti-Rabbit IgG and/or TRITC Goat Anti-Mouse, IgG), BODIPY to stain lipid droplets and DAPI to stain the nuclei, for 2 h at room temperature.

### Confocal imaging

A confocal laser-scanning microscope was used to image samples (Leica TCS SP8 Confocal Microscope; Buffalo Grove, IL). Adipocytes labeled by BODIPY and ITA7 labeled by Alexa Fluor 647 were imaged simultaneously in two channels, with Rhodamine-labeled endothelial tissue or TRITC CD68-labeled macrophages, and DAPI nuclei imaged in series. Confocal image stacks were obtained with a 63 × oil immersion lens (NA 1.4). Laser intensity, confocal aperture, and photomultiplier gain were kept constant across samples.

*Image processing and analysis:* Confocal image stacks were processed and analyzed using Leica LASX software version 3.5.2 (Wetzlar, Germany). The Alexa 647 and Rhodamine channels were thresholded and the intersection between both layers was quantified in order to characterize the colocalization between ITA7 and endothelial cells. In separate tissue, the colocalization between ITA7 and CD68 positive cells was also quantified. Using 3D images, the Alexa 647, Rhodamine or TRITC and intersection area per cell were calculated.

*cytoNet image analysis:* cytoNet is a software accessible over the web that quantifies the spatial relationships in cell communities using principles of graph theory, and evaluates the effect of cell–cell interactions on individual cell phenotypes (https://www.braininitiative.org/toolmakers/resources/cytonet/). Image files were segmented manually and provided as input to cytoNet. Because blood vessels have noncircular shapes, the minimum distance between object perimeters was computed in order to define graph edges. The resulting cell-to-cell perimeter distance and cells characteristics were analyzed in WT and KO SubQ WAT images.

### RNA isolation and quantitative RT-PCR

RNA from tissue and cells was isolated and purified using a Qiagen RNeasy Mini Kit (Valencia, CA) according to manufacturer guidelines. mRNA concentrations were measured using a Take3 Micro-Volume Plate (BioTek, Winooski, VT), then normalized to 150 ng of mRNA for conversion to cDNA. cDNA was synthetized using random hexamer primers and an iScript cDNA-synthesis kit (Biorad). The quantitative RT-PCR reactions were performed using the So Advanced™ Universal SYBER Green Supermix kit (Biorad) in a CFX96 Touch Real-Time PCR Detection System (BioRad, Hercules, CA). Fold expression levels were calculated using the 2^−∆∆Ct^ method. Transcript levels were normalized to 18S ribosomal RNA levels in all experiments that used mice cells (Figs. 3,4,7), or the GAPDH reference gene in experiments with human cells (Fig. 6). Primer (Table 1) specificity was tested by the assessing the melting curve.

**Table 1:** Quantitative real-time polymerase chain reaction primer sequences.

Primer	Forward primer	Reverse primer
18 s (m)	5′-GTAACCCGTTGAACCCCATT	5′-CCATCCAATCGGTAGTAGCG
UCP1 (m)	5′-ACTGCCACACCTCCAGTCATT	5′-CTTTGCCTCACTCAGGATTGG
ITA7 (m)	5′-GATCGTCCGAGCCAACATCACA	5′-CTAACAGCCCAGCCAGCACT
Adiponectin (m)	GAATCATTATGACGGCAGCA	TCATGTACACCGTGATGTGGTA
COL1A1 (m)	CGTATCACCAAACTCAGAAG	GAAGCAAAGTTTCCTCCAAG
COL3A1 (m)	5′-CTGGAGAACCTGGTGCAAAT	5′-CCTCGGAAGCCACTAGGAC
ITB1 (m)	AATGTTTCAGTGCAGAGCC	TTGGGATGATGTCGGGAC
LAMA4 (m)	5′-GGAATACCTGAACGTGCACATGAGA	5′-GTGCCATCTGCCATCACAGAGATTCT
GAPDH (h)	5′-TGACAACTTTGGTATYCGTGGAAGG	5′-AGGCAGGGATGATGTTCTGGAGAG
UCP1 (h)	5′-TCTACGACACGGTCCAGGAG	GAATACTCCCACTCCTCCAGTC
LAMA4 (h)	5′-GCGGCCGAGAAATGCA	5′-AGTCGCAGGGCACACATTC
ITA7 (h)	5′-ACCAATACCCTGACCTGCTG	5-CTATAGCTGCTGGGGACTGC
Adiponectin (h)	5′-AAGGAGATCCAGGTCTTATTGG	5′-ACCTTCAGCCCCGGGTAC

### Western blot analysis

Cells were lysed using CelLytic (Sigma, St. Louis, MO) and tissue was lysed using RIPA buffer (EMD Millipore, Billerica, MA) and protease inhibitor cocktail (Fischer Scientific, Waltham, MA). Protein concentration was determined using the Bio-Rad DC protein assay (Bio-Rad Laboratories, Hercules, CA). Samples were diluted in water and loading buffer (LI-COR, Lincoln, NE), electrophoretically separated under denaturing conditions on 4–15% SDS-PAGE Criterion gels (Bio-Rad), and transferred to nitrocellulose membranes (Bio-Rad). Membranes were blocked in Intercept Protein-Free Blocking Buffer (LI-COR), followed by an overnight incubation with primary antibody for UCP1 (Invitrogen, Carlsbad, CA; rabbit polyclonal, #PA1-24894), ITA7 (Novus, Centennial CO; rabbit polyclonal, #NBP1-74207) or GAPDH (Santa Cruz; mouse monoclonal, #sc-32233) diluted in Intercept® Protein-Free Antibody Diluent (LI-COR). Membranes were incubated with appropriate IRDye secondary antibodies (LI-COR), and immunodetection was performed using near-infrared Odyssey Fc Imaging System (LI-COR). Images were obtained with Image Studio Software (LI-COR).

### Cell culture primary adipose-derived stem cells

*Isolation*: SubQ adipose depots were harvested from euthanized KO and WT mice and digested in collagenase type I. The adipose-derived stem cells were enzymatically isolated following a previously published protocol^55^. Briefly, digestion was performed in an orbital shaker at 37 °C for 60 min. The digest was then centrifuged and the floating adipocyte layer discarded. The pelleted stromal vascular fraction was washed two times with complete media (CM, Dulbecco’s modified Eagle’s Ham’s F12 medium supplemented with 10% fetal bovine serum, and 1% antibiotic–antimycotic) and plated on tissue culture plastic. Cells at passages 2 to 5 were used for experiments.

*Adipose derived stem cells beige differentiation:* Cells (passage 2–4) were cultured in 24 well tissue culture plates and incubated in CM. When cells reached 95% confluence differentiation was initiated. For beige differentiation cells were incubated for 4 days in induction media (CM with 5 μg/ml insulin, 10 μM forskolin, 2 μg/ml dexamethasone, 125 μM indomethacin, 0.5 μM Rosiglitazone and 1 nM triiodothyronine) and then maintained in CM with 5 μg/ml insulin, 10 μM forskolin, 1 μM rosiglitazone and 1 nM triiodothyronine. Differentiation media was changed every other day until harvest.

*siRNA transfection:* Adipocytes were transfected at day 10 of differentiation. The lipofectamine RNAiMAX preparation was carried out following the manufacturer’s instructions. For a 24 well plate, the lipofectamine RNAiMAX reagent (3 μl per well) and the siRNA (10 μM) were diluted separately in serum free media and mixed by pipetting. The siRNA-RNAiMAX mix was left to incubate for 5 min at room temperature after which the siRNA-RNAiMAX mix was added on top of the adherent cells. Adipocytes were transfected twice during the differentiation process and harvested 3 d after the last transfection (14 days after differentiation).

*Surface coated plates:* Collagen 3A1, Collagen 1A1 and Laminin α4 recombinant proteins (R&D Systems) were reconstituted in PBS to obtain a working concentration of 20 µg/ml. 24 wells—tissue culture plates were coated with 300 μl of each reconstituted protein and incubated for 4 h at 37 °C. Excess fluid was removed and the plates allowed to dry before introducing cells and media. All protocols were performed under sterile conditions.

### Hydrogen peroxide assay

The ROS-Glo H2O2 Assay uses a modified luciferin substrate, based on boronate oxidation, which reacts directly with hydrogen peroxide (H2O2) to generate a luciferin precursor. Upon addition of detection reagent, precursor is converted to luciferin and Ultra-Glo Recombinant Luciferase included in the detection reagent produces a light signal proportional to the level of H2O2 in the sample. Briefly, ASCs were differentiated in LAMA4 coated surfaces or uncoated surfaces in 48 wells. Negative control cells were not differentiated and kept in growth media. 6 h before the end of the differentiation 150 µl of a mixture of H2O2 substrate and H2O2 dilution buffer was added to each well. Six hours later, 50 µl of the media was mixed with 50 µl of ROS-GLO Detection Solution in a separate plate and incubated at room temperature for 20 min. Luminescence was read by Take3 Micro-Volume Plate (BioTek, Winooski, VT).

### Cell culture and differentiation of human adipose derived stem cells

Commercially available hASCs (PT-5006, Batch 0,000,535,975, Lonza) were used at passage 2 or 3. Cells were seeded at a density of 25,000 cells/cm^2^ in CM. The media was changed every other day. When cells reached confluence differentiation was initiated (designated as day 0). Cells were then maintained in Dulbecco’s modified Eagle’s Ham’s F12 (DMEM/F12) media supplemented with 850 nM insulin, 250 µM isobutyl-methylxanthine, 125 nM indomethacin, 0.5 μM dexamethasone, 1 µM rosiglitazone and 120 nM triiodothyronine. Differentiation media was changed every other day.

### Statistical methods

Graphpad Prism Software 6 (GraphPad Software, Inc., La Jolla, CA) was used to run unpaired T tests with Welch's correction and one way analysis of variance (ANOVA) tests with Tukey or Dunnet multiple comparison analysis (as specified in each figure) to determine differences between groups. Statistical significance was defined when *p* < 0.05. All results are presented as mean ± standard error of the mean (SEM).

## Supplementary Information

Supplementary Information

## Data Availability

All data generated or analysed during this study are included in this paper.
