# Supplementary material for: Integrins and extracellular matrix proteins modulate adipocyte thermogenic capacity
Source: Sci Rep. 2021 Mar 8;11:5442. doi: 10.1038/s41598-021-84828-z (PMC7940610; doi:10.1038/s41598-021-84828-z)

**SUPPLEMENTARY INFORMATION**

**Integrins and extracellular matrix proteins modulate adipocyte thermogenic capacity**

Maria A. Gonzalez Porras^1^, Katerina Stojkova^1^, Marcella K Vaicik^2^, Amanda Pelowe^3^, Anna Goddi^4^, Alanis Carmona^4^, Byron Long^1^, Amina A. Qutub^1^, Anjelica Gonzalez^3^, Ronald N Cohen^4^ and Eric M. Brey^1^

^1^Department of Biomedical Engineering and Chemical Engineering, The University of Texas at San Antonio, San Antonio, TX. ^2^Department of Biomedical Engineering, Illinois Institute of Technology, Chicago, IL. ^3^Department of Biomedical Engineering, Yale University, New Haven, CT. ^4^Section of Endocrinology, Diabetes, and Metabolism, Department of Medicine, University of Chicago, Chicago, IL.,

**Supplemental Figure 1.** Representative low magnification confocal images of CD68 and ITA7 levels and spatial distribution in KO and WT adipose tissue. Immunohistochemical 20x confocal images of fixed adipose tissue from KO mice and WT mice. Formaldehyde-fixed tissues were stained with antibodies against CD68 (red) and ITA7 (yellow). Endothelial cells were counterstained with BODIPY (green) and the nucleus with DAPI (blue).

**
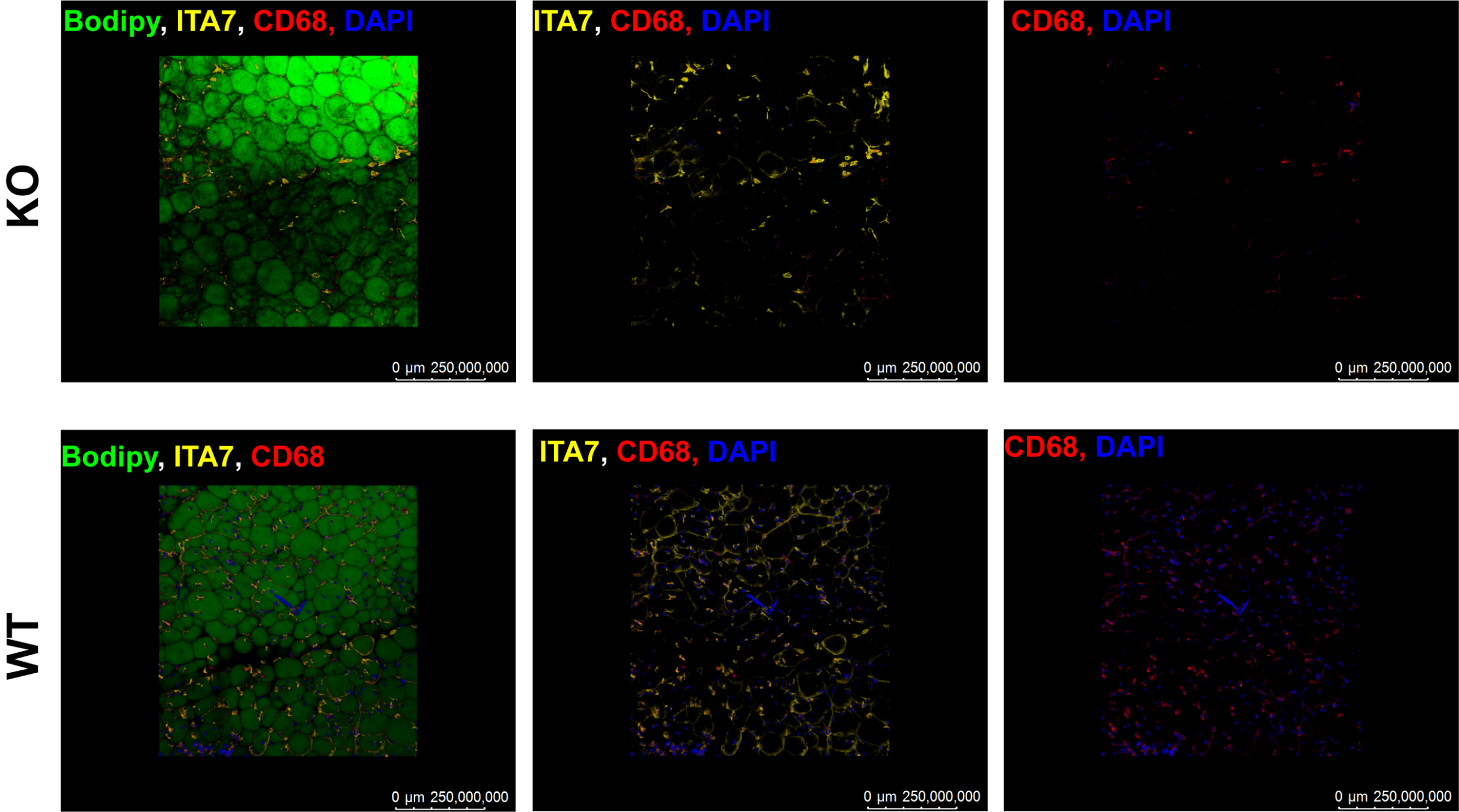
**

**Supplemental Figure 2.** Analysis of CD68 levels in KO and WT adipose tissue. (A) Higuer magnification representative 3D images of CD68 and ITA7 cells in the stromal space of WT adipose tissue. (B) CD68 volume was lower in KO tissue compared to WT (p<0.0001). (C) There is not significant difference in the percent of CD68 cells colocalized with ITA7 between KO and WT adipose tissue (p=0.17). Values are mean ± standard error from unpaired t test with Welch's correction.

**
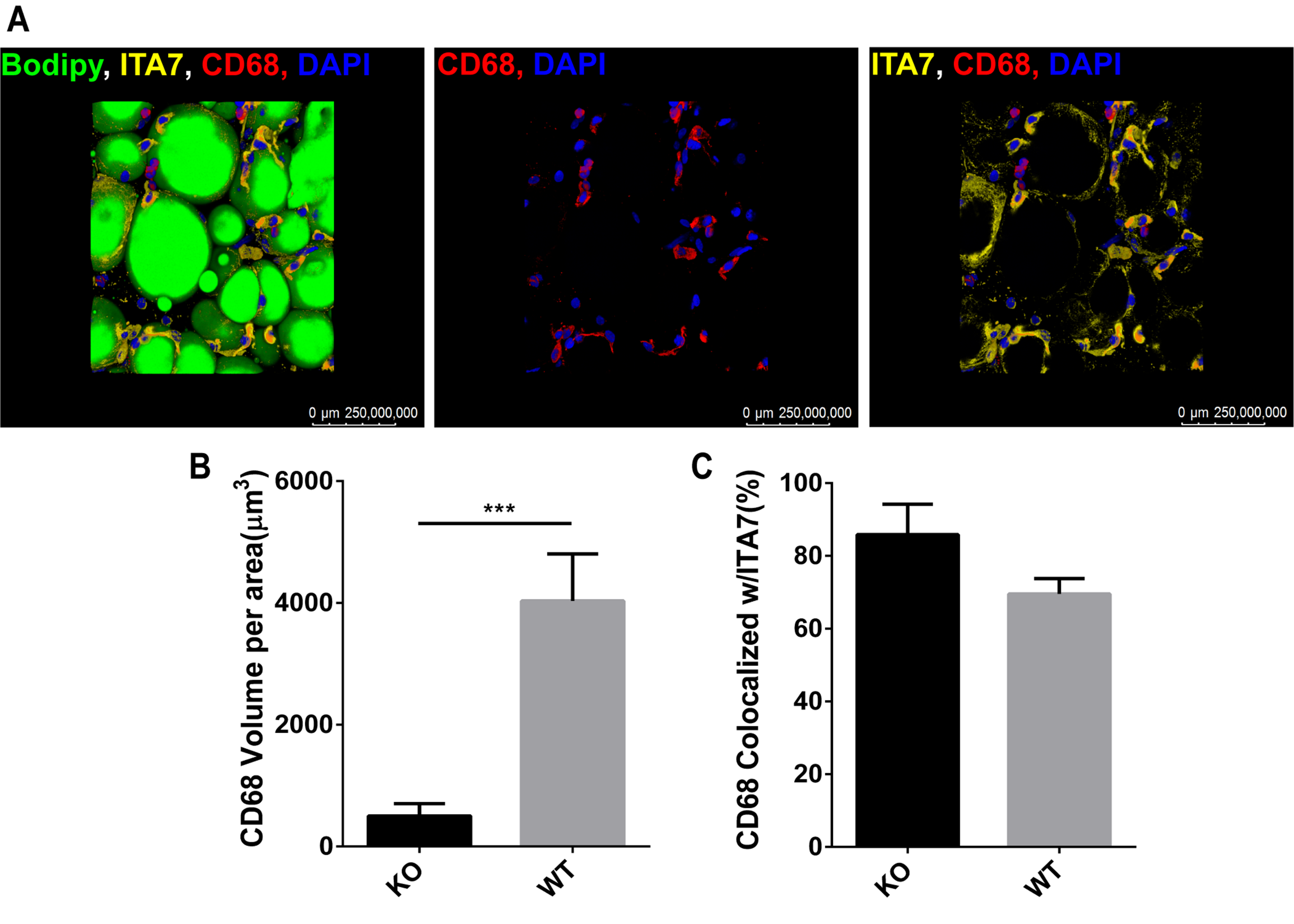
**

**Supplemental Figure 3.** Timeline mRNA levels of beige markers PGC1A, COX7A1, CIDEA and DIO2 during beige differentiation of hASCs. Values are means ± standard errors, from one-way ANOVA analysis followed with Tukey's multiple comparisons test. **, p < 0.01; ***, p < 0.001; n=2 different experiments with 4 replicates per group per experiment.

**
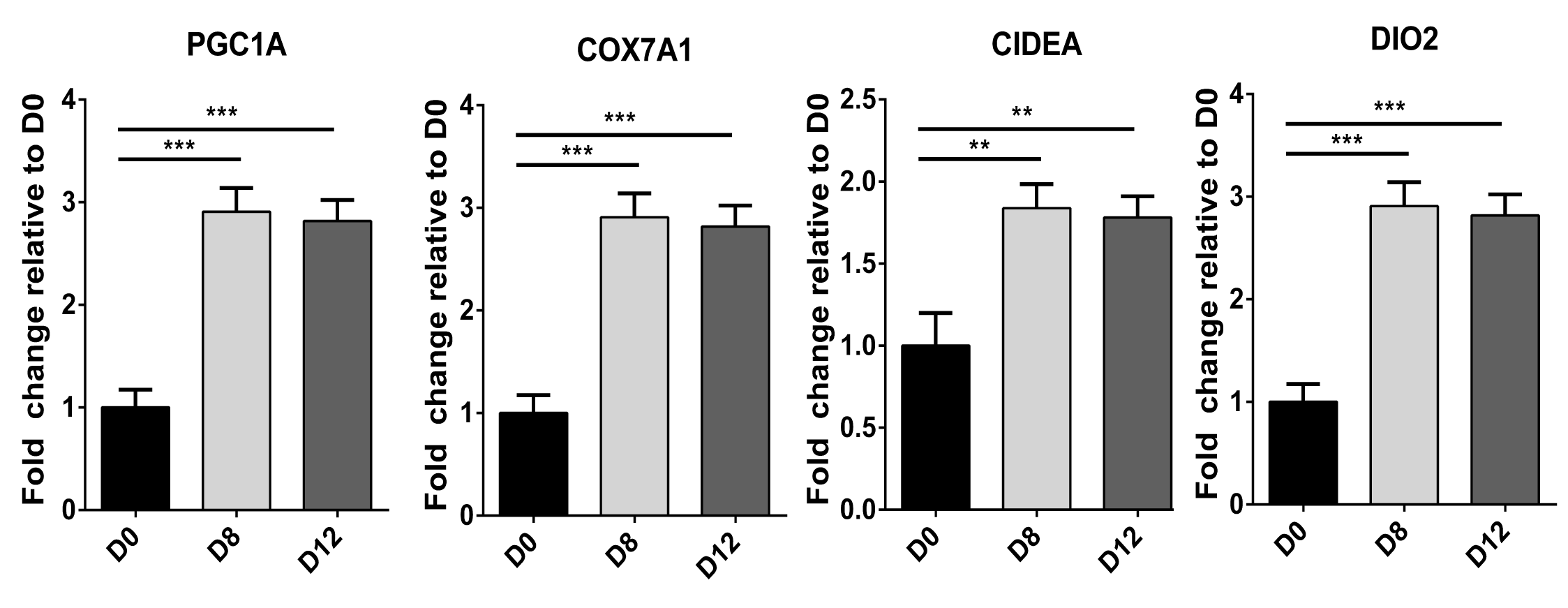
**

**Supplemental Figure 4.** Schematic illustration of therapeutic application. ITA7 modulates the metabolic function of the adipocytes. This information identifies ITA7 as a new therapeutic target for obesity and metabolic diseases. Manipulating levels of ITA7 in adipose tissue could improve systemic energy metabolism and glucose homeostasis. Similarly, lowering levels of CO3A1 and CO1A1 in SubQ adipose tissue may create an environment that helps promoting differentiation of stem cells into beige adipocytes.


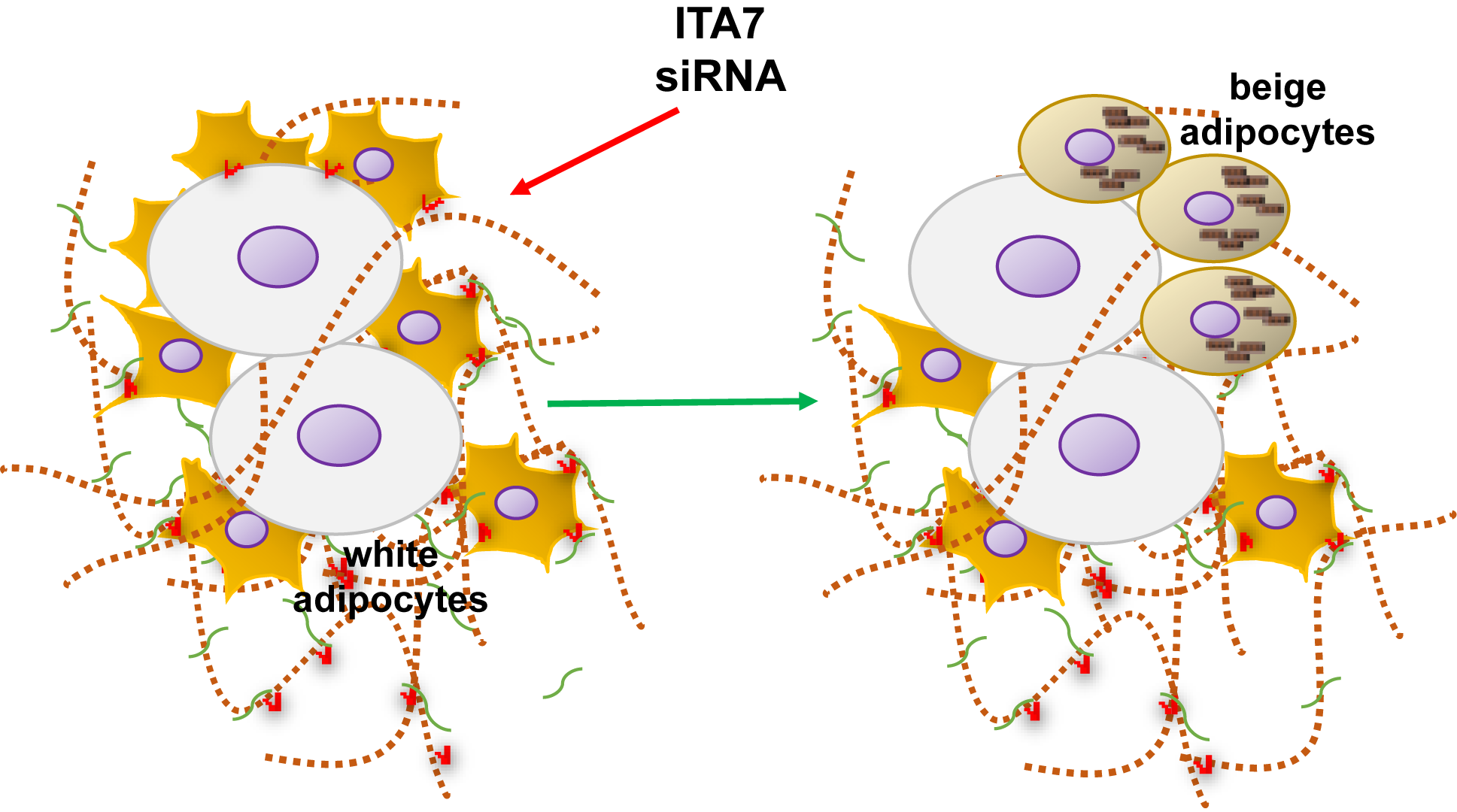


**Supplemental Figure 5.** Uncropped western blots from Figure 4H. (A) Detection of UCP1 and GAPDH (mirrored in figure). (B) Detection of ITA7 and GAPDH (mirrored in figure)


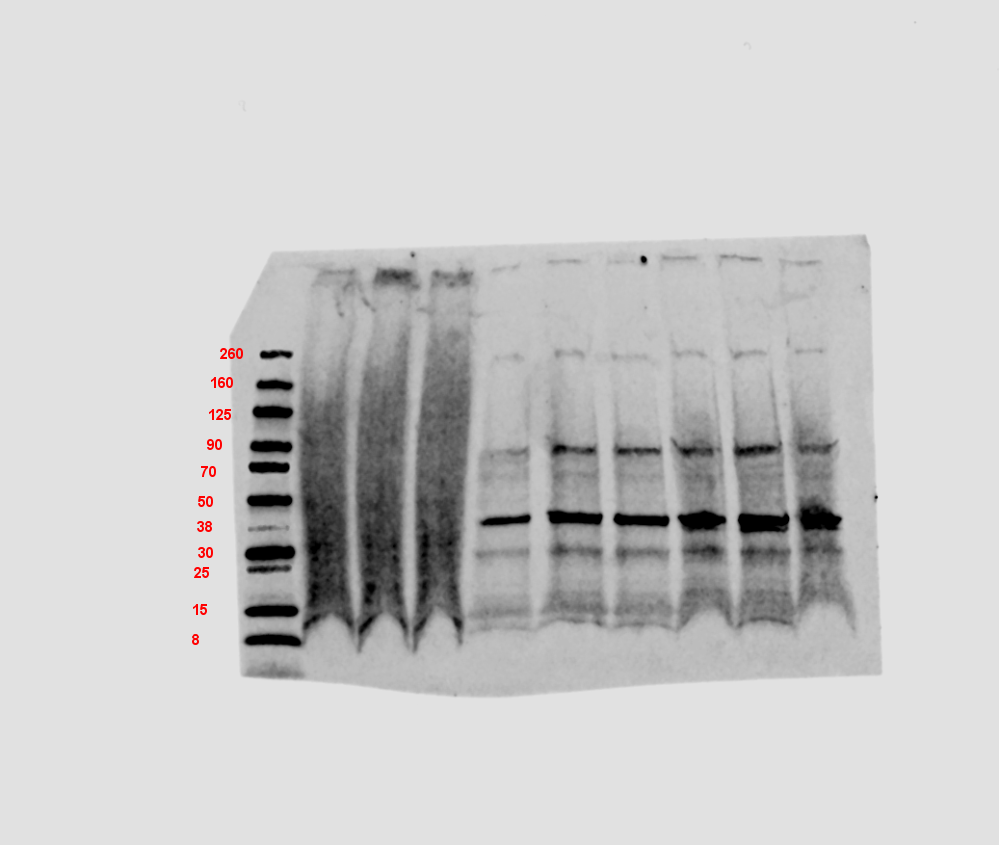


**A**


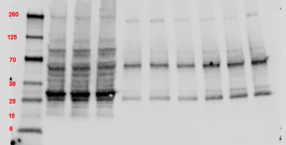


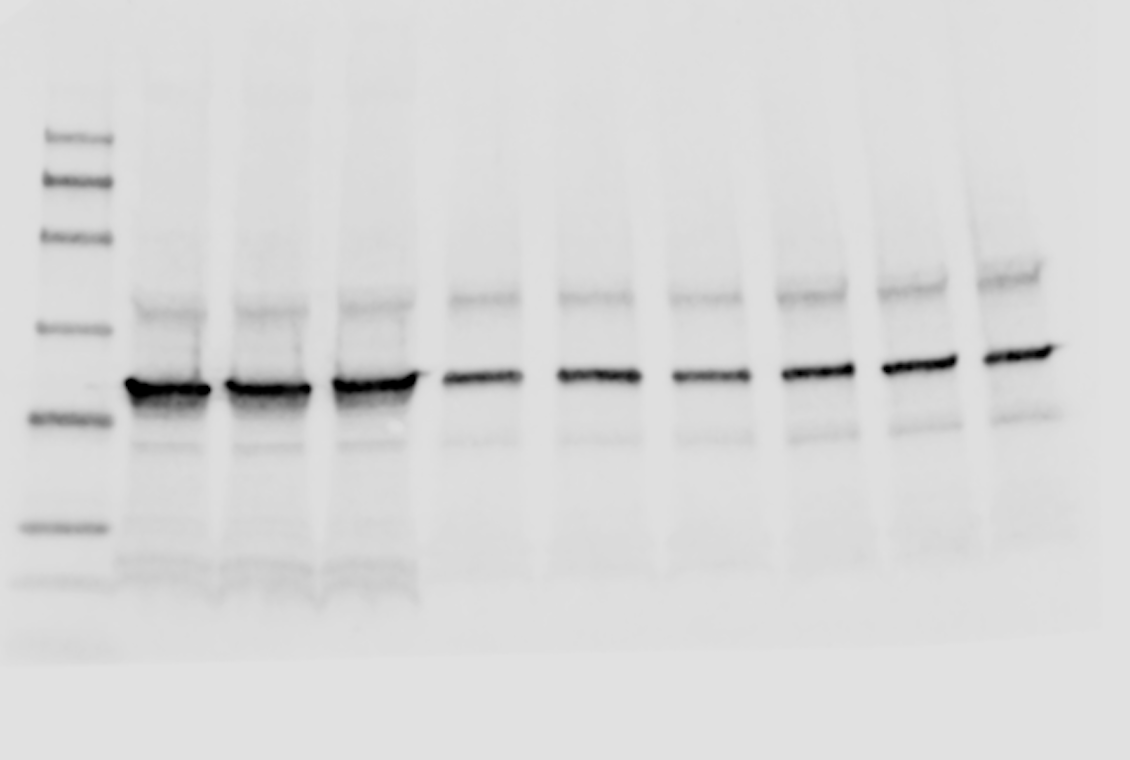


**B**


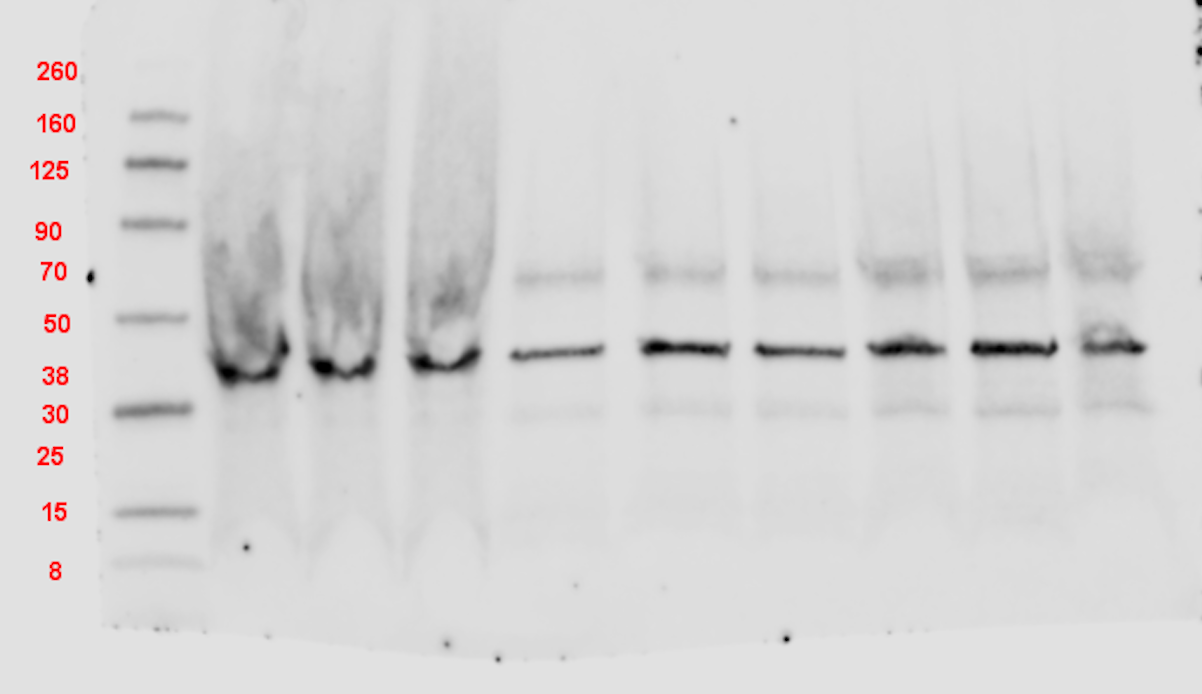

Supplement: Supplementary file 1 — Supplementary Information [file 41598_2021_84828_MOESM1_ESM.docx]
